# Effects of different selenium fertilizer types and dosages on non-volatile organic acids and aroma substances of flue-cured tobacco

**DOI:** 10.3389/fpls.2025.1659004

**Published:** 2025-08-22

**Authors:** Wanhui Jiang, Zijun Sun, Huaiyuan Li, Yaoxing Liang, Qihang Yang, Xin Yang, Lanjun Shao, Yuanyuan Wang, Jianjun Chen, Shiyuan Deng

**Affiliations:** ^1^ College of Agriculture, South China Agricultural University, Guangzhou, China; ^2^ Center for Basic Experiment and Practice Training, South China Agricultural University, Guangzhou, China; ^3^ China Tobacco Guangdong Industrial Co., Ltd., Guangzhou, China

**Keywords:** selenium, flue-cured tobacco, non-volatile organic acids, aroma precursors substance, neutral aroma substance

## Abstract

Tobacco (*Nicotiana tabacum* L.) is well-known as an economic crop whose quality is evaluated according to its aroma quality. Researchers have found that selenium application can increase the aroma quality of tobacco, but until now, its mechanism is still unclear. So a field experiment was conducted to investigate the regulatory effect of the trace element selenium on enhancing aroma substances in flue-cured tobacco leaves. We compared selenium content, aroma precursors, non-volatile organic acids (NVOAs) content, and neutral aroma substances in tobacco upper leaves that applying three selenium fertilizer types (sodium selenite, organic selenium and nano-selenium) with three levels of pure selenium application rates (5.24 g·hm^-2^, 10.48 g·hm^-2^, and 15.72 g·hm^-2^) through foliar spraying. The results showed that the neutral aroma substances and NVOAs content were significantly increased. Metabolomics analysis of NVOAs showed that biosynthesis of phenylpropanoids pathway was significantly enriched. This indicates that selenium application may be able to regulate the accumulation of phenylpropane degradation products by regulating these NVOAs involved in the biosynthesis of phenylpropanoids pathway. Selenium content was significantly positively correlated with phenylpropane degradation products, NVOAs content, and phenolic compounds, while pyruvate was significantly down-regulated in each selenium application treatment. Therefore, it was speculated that selenium application could enhance the conversion of pyruvate into the biosynthesis of phenylpropanoids pathway. In summary, spraying organic selenium at 5.24–15.72 g·hm^-2^ or nano-selenium at 5.24–10.48 g·hm^-2^ could provide a suitable strategy for improving tobacco aroma quality.

## 1 Introduction

Tobacco is well-known as an economic crop, valued for its aroma quality, its fragrance type and quality depends on the content of aroma substances and is indirectly affected by the content and components of organic acids (Yun et al., 2013; Zou et al., 2023). In flue-cured tobacco, organic acids make up 12–16% of the total composition, with significant differences among varieties (Xiang et al., 2010). Based on their physical characteristics, organic acids can be classified into volatile, semi-volatile, and non-volatile acids(NVOAs), with NVOAs accounting for the largest proportion (Jing et al., 2013). These organic acids constitute up to 7% of the total weight of tobacco leaves (Yun et al., 2013) and are considered part of the latent aroma components of tobacco (Fan et al., 2022). They can combine with alkaline substances to form salt, adjust the proportion of free nicotine, mellow the taste, and increase smoke concentration. In addition, they can influence the stability of certain aroma substances, thereby indirectly affecting the aroma quality (Wang et al., 2008; Lin et al., 2018; Tie et al., 2024).

Researchers have proved that organic acids in plants can further affect carbon and nitrogen metabolism and the biosynthesis of secondary metabolites by participating in amino acids and sugars synthesis (Medeiros et al., 2021). The aroma quality of tobacco depends on many secondary metabolites, which are ultimately originate from photosynthesis, glycolysis, the tricarboxylic acid (TCA) cycle, the methylerythritol phosphate pathway (MEP) and amino acid metabolism (Fiesel et al., 2022). And phenolic acids within organic acids, such as quinic acid, shikimic acid, and chlorogenic acid, are involved in browning reactions and shikimic acid metabolism. Their downstream phenylalanine degradation reactions can form a large number of volatile aroma substances (Williamson and Gwynn, 1982; Zhao et al., 2022, 2023; Xu et al., 2024), which are the main source of sensory quality formation in tobacco (Wang et al., 2018).

Some organic acids are significantly related to the sensory quality of tobacco, which has been explored by many researchers. For example, the correlation analysis of NVOAs and sensory quality showed that citric acid had a positive impact on the aroma and the score of impurities in tobacco leaves (Wang et al., 2018). Additionally, the aroma discrimination function of flue-cured tobacco based on organic acids, amino acids, and aroma substances has been established (Wang et al., 2024), indicating that the content of certain organic acids plays an important role in the differences in the fragrance type of flue-cured tobacco. The infrared speed measurement model established (Fu et al., 2024), which analyzed NVOAs, higher fatty acids, conventional chemical components, and sensory evaluation indexes in the upper leaves of flue-cured tobacco, identified palmitic acid, malic acid, and fumaric acid as important indexes for evaluating the sensory characteristics of upper leaves. Although many studies have confirmed the effect of organic acids on the sensory quality of flue-cured tobacco through their participation in balancing the taste of flue gas, few studies explored how NVOAs metabolically influence the formation of neutral aroma substances. Therefore, it is of great significance to explore how to enhance the synthesis of aroma substances in tobacco by implementing agronomic measures to control organic acid components and content during production.

Selenium is an important plant nutrient element that has been well-documented for its effectiveness in enhancing plant stress resistance (Di et al., 2025; Dai et al., 2019), improving photosynthetic capacity (Jiang et al., 2015), and promoting plant growth (Wang et al., 2012). Its effects on plants are dual: at low concentrations, it has a positive effect on the growth and development of plants, while at high concentrations, it shows an inhibitory effect (Hartikainen et al., 2000; Jiang et al., 2015; Han et al., 2013; Zhang et al., 2023). As an active site of glutathione peroxidase and other enzymes, selenium can participate directly or indirectly in free radical scavenging and inhibit membrane lipid peroxidation (Huang et al., 2024). It enhances plant resistance to cold, high temperature, drought, and heavy metals by activating the antioxidant oxidase system (Bai et al., 2024; Saleem et al., 2020; Dai et al., 2023; Jia et al., 2023).

Studies had shown that the application of low doses of selenium can promote the synthesis of proteins related to photosynthesis and carbon and nitrogen metabolism, enhance the activity of related metabolic enzymes (Wang et al., 2012; Cunha et al., 2022), improve the net photosynthetic rate of fragrant rice, increase the seed setting rate at maturity, and ultimately achieve the goal of increasing production (Luo et al., 2020). In recent years, there have been some reports on the application of selenium in crops to enhance fragrance. For example, applying selenium to the leaf surface can significantly improve the non-volatile and volatile components of tea, resulting in a sweet aftertaste and nectar-like aroma (Xu et al., 2024). Applying exogenous selenium during the early heading stage can increase the content of 2-AP-related synthetase and its synthetic precursor of fragrant rice (Luo et al., 2020). Selenium can also promote the conversion of pyruvate to acetyl-CoA by regulating enzyme activities involved in metabolic pathways such as glycolysis and the tricarboxylic acid (TCA) cycle in broccoli. It further promotes the conversion of acetyl-CoA into volatile aroma substances such as aldehydes and esters and induces transcription factors related to aroma formation (Liu et al., 2023). However, this regulatory mechanism remains unclear in tobacco. Although a large number of studies have shown that selenium application can increase the content of NOVAs and volatile aromatic substances in crops, the regulatory effects of NVOAs and formation of volatilearoma substances have not yet been linked. In response to this, for the first time, we used LC-MS/MS to link NVOAs with the response of phenylpropanoid biosynthesis in tobacco to selenium.

In a previous study, we found that selenium foliar application can effectively increase the content of aroma substances in flue-cured tobacco. Building on that, we further explored the effects of selenium application on NVOAs in flue-cured tobacco. Through metabolomics analysis and correlation analysis, we investigated the mechanism by which NVOAs influence aroma substances in flue-cured tobacco. Additionally, we compared the effects of sodium selenite, organic selenium fertilizer, and nano-selenium fertilizer on enhancing aroma substances, aiming to provide a theoretical basis for the application of exogenous selenium to increase selenium content and improve aroma in tobacco production.

## 2 Materials and methods

### 2.1 Experimental site description

The tobacco cultivar Yunyan 87 used in this experiment was provided by the Hengyang Branch of Hunan Provincial Tobacco Company and cultivated at He Xi Village (112°50^′^50^′′^ E, 26°43^′^19^′′^ N) in Hengnan County, Hengyang City, Hunan Province, with an average precipitation from 144.04 to 373.13 mm and average temperature ranging from 16.11 to 32.12 °C during the tobacco growing season in 2024 (**Supplementary Figure S1**). The soil texture of the study site there was red clay loam. And the chemical characteristics of the study soil as followed: 46.43 g·kg^-1^ of organic matter, 2.35 g·kg^-1^ of total nitrogen, 2.25 g·kg^-1^ of total potassium, 0.82 g·kg^-1^ of total selenium, 102.22 mg·kg^-1^ of alkali-hydrolyzable nitrogen, 5.50 mg·kg^-1^ of rapidly available phosphorus, 393.85 mg·kg^-1^ of rapidly available potassium, and a pH of 6.7. The fertilization rate was set at 900 kg·hm^-2^, with a ratio of m (N): m (P2O5): m (K2O) maintained at 8: 10: 11.

### 2.2 Experimental design

In the previous studies in 2023, we have found that when application amount of organic selenium is greater than 10.50 g·hm^-2^ (**Supplementary Table S1**), the selenium content on the leaf surface after flue-curing (**Supplementary Table S2**) exceeds the appropriate selenium content proposed in the existing studies (Cao et al., 2017). Based on this result, the application amounts of sodium selenite and nano- selenium fertilizer were set in reference to the application amounts of organic selenium fertilizer in 2023, 10 treatments were set up, which were divided into three types of selenium fertilizer and three levels as shown in **Table 1**. All treatments were conducted in a randomized block design with 3 biological replicates.

**Table 1:** Experimental treatment.

Treatment	Selenium fertilizer type	Single application	Total selenium application
CK	Water only	none	none
SS1	sodium selenite	2.62 g·hm^-2^	5.24 g·hm^-2^
SS2	sodium selenite	5.24 g·hm^-2^	10.48 g·hm^-2^
SS3	sodium selenite	7.86 g·hm^-2^	15.72 g·hm^-2^
OS1	organic selenium fertilizer	2.62 g·hm^-2^	5.24 g·hm^-2^
OS2	organic selenium fertilizer	5.24 g·hm^-2^	10.48 g·hm^-2^
OS3	organic selenium fertilizer	7.86 g·hm^-2^	15.72 g·hm^-2^
NS1	nano-selenium fertilizer	2.62 g·hm^-2^	5.24 g·hm^-2^
NS2	nano-selenium fertilizer	5.24 g·hm^-2^	10.48 g·hm^-2^
NS3	nano-selenium fertilizer	7.86 g·hm^-2^	15.72 g·hm^-2^

The tobacco seedlings used in the experiment were transplanted on March 15, 2024. Selenium fertilizer was evenly sprayed twice during the growth period, respectively at the rosette stage (45d after transplanting, April 28, 2024) and vigorous growth stage (60d after transplanting, May 13, 2024). Spraying is carried out on a clear and cloudless morning, ensuring that both sides of all leaves on the plant were evenly sprayed. The inorganic selenium used in the test was pure sodium selenite, which was purchased from Shanghai Aladdin Reagent Company. Organic selenium, known as selenium-rich treasure, containing 7 g·L^-1^ selenium, was provided by Guangdong Guanlv Fertilizer Co., LTD. Nano-selenium was a bionnanometer selenium nutrient planting solution with a selenium content of 5 g·L^-1^, purchased from Wuhan Hua Selenium Biotechnology Co., LTD. Each treatment replicated in three plots in this experiment, with a randomized block design adopted. And 30 plots were established in total, each with a row spacing of 1.2 m and a plant spacing of 0.45 m as conducted. Each plot contained two rows with 35 plants per row, totaling 70 plants per plot. Around the experimental field, two lines of tobacco plant protection were established. Field management measures are implemented in accordance with the local production plan.

### 2.3 Measurement parameters and methods

#### 2.3.1 Chemical and sample

The selenium standard was provided by the Test Center of South China Agricultural University. The nitric acid and perchloric acid used for sample digestion were of high purity and were purchased from Shanghai Aladdin Biochemical Technology Co., LTD. All of the commercial standards (chromatographic grade, ≥ 99% purity) used to determine NVOAs and neutral aroma substance were purchased from Sigma-Aldrich (St. Louis, MO, USA).

After flue-curing, the upper tobacco leaf samples (B2F) were sent to laboratory for further research, and each sample was set up with three replicates. The samples collected were dried at 40°C, ground into powder with a grinder, and then sifted (pore size: 0.25 mm) for determination of selenium content, petroleum ether extract, phenolic compounds, plastid pigments, non-volatile organic acid content, and neutral aroma substances.

#### 2.3.2 Selenium content

The selenium content of samples was measured with a Graphite furnace atomic absorption spectrometer (Jena, ZEEnit650P), following the corresponding state criteria for tobacco and tobacco products of China (YC/T 221—2007). The collected samples were digested with a 4:1 volume ratio of nitric acid to perchloric acid and analyzed using an atomic fluorescence spectrophotometer.

#### 2.3.3 Petroleum ether extract

The determination was conducted with reference to the criteria for tobacco and tobacco products of China (YC/T 176-2003) and the improved method of Shao et al (Shao et al., 2010). A total of 2.000g of tobacco powder was weighed (M_0_) and placed into a filter paper cartridge. The sample was dried at 80.0°C for 2 h, immediately cooled in a desiccator for 30 min, and then weighed as M_3_. The dried filter paper cartridge was transferred to a Soxhlet extractor, and 300 mL petroleum ether was added, which was then left at room temperature overnight. The next day, three zeolite and 150 mL of petroleum ether were added to the receiving bottle for reflux extraction, which was carried out in a water bath maintained at 75°C for 6 h. Upon completion of the extraction, the filter paper cartridge was dried at 80.0°C for 2 h, then cooled in a desiccator for 30 min, and weighed as M_4_. And an empty filter paper cartridge was performed simultaneously as. The total amount of petroleum ether extract was calculated using the following Equation 1:


(1) $$\text{Total petroleum ether extract }\left(\%\right)=\frac{\left(M_{1}-M_{2}\right)-\left(M_{3}-M_{4}\right)}{M_{0}\times \left(1-W\right)}$$

Where M_1_ and M_2_ are the combined weights of the sample and filter paper cartridge before and after extraction, respectively (g). M_3_ and M_4_ are the weights of the filter paper cartridge before and after extraction, respectively (g). M_0_ is the net weight of the sample, and *W* is the moisture content of the sample (%).

#### 2.3.4 Phenolic compounds content

The total phenol content was determined using the microplate method with a commercially available assay kit, purchased from Suzhou Gris Biotechnology Co., LTD, with the microplate method. Each sample was measured in 3 times.

#### 2.3.5 Plastid pigments content

The plastid pigments content, including chlorophyll a, chlorophyll b, total chlorophyll (the sum of chlorophyll a and chlorophyll b), and carotenoids, was determined by spectrophotometry using a commercially available assay kit purchased from Suzhou Gris Biotechnology Co., LTD. Each sample was measured in 3 times.

#### 2.3.6 NVOAs extraction

The composition and content of NVOAs were detected by MetWare (http://www.metware.cn/) based on the AB Sciex QTRAP 6500 LC-MS/MS platform. Weighed 50 mg of the sample powder and placed into a 2 ml centrifuge tube. A total of 850 μL of the internal standard extraction solution, pre-cooled at -20°C (methanol: acetone: water = 7:7:3), was added to the tube and swirled for 3 min. The sample was centrifuged at 12000 r/min for 10 min at 4°C. The supernatant was then placed at -20°C for 30 min before being centrifuged again for 10 min under the same centrifuge conditions. Next, 200 μL of the supernatant was added to 200 μL of the 70% methanol-water internal standard extraction solution and mixed for 1 min. The 200 μL Protein Precipitation Plate was obtained by passing the mixture through a nylon filter membrane and used for on-machine analysis.

#### 2.3.7 NVOAs analyses by UPLC-MS/MS

The acquisition of data is mainly carried out through Ultra Performance Liquid Chromatography (UPLC) and Tandem Mass Spectrometry (MS/MS). The HPLC column used was an ACQUITY HSS T3 column (1.8µm, 100 mm×2.1 mm i.d.). The mobile phase consisted of phase A: ultra-pure water (0.05% formic acid), and phase B: acetonitrile (0.05% formic acid). The gradient elution procedure was as follows: 95:5 (V/V) for A/B at 0 min, 5:95 (V/V) for 8–9.5 min, and 95:5 (V/V) for 9.6–12 min. The flow rate was 0.35 mL/min, with a column temperature of 40°C, and the sample size was 2 μL. Mass spectrometry conditions were as follows: an Electrospray Ionization (ESI) temperature of 550°C, mass spectrum voltage of 5500 V in positive ion mode, -4500 V in negative ion mode, and Curtain Gas (CUR) at 35 psi. In the Q-Trap 6500+ system, each ion pair was scanned against an optimized declustering potential (DP) and collision energy (CE). A Metware Database (MWDB) for NVOAs was constructed based on standard products, and qualitative analysis of mass spectrometry data was performed using Analyst 1.6.3 software. Quantitative analysis was conducted using Multiple Reaction Monitoring (MRM) with triple quadrupole mass spectrometry. After obtaining mass spectrometry data from different samples, chromatographic peaks of all target objects were integrated, and quantitative analysis was performed using standard curves.

#### 2.3.8 Neutral aroma substance

The identification and quantification of neutral aroma substance was carried out using an Agilent Model 8890 GC and a 7000D mass spectrometer (Agilent), equipped with a 30 m × 0.25 mm × 0.25 μm DB-5MS. The carrier gas was high purity helium (purity ≥99.999%), with a constant flow rate of 1.2 mL/min. The inlet temperature was 250°C, with no split injection, and a solvent delay of 3.5 min. Mass spectra was recorded in electron impact (EI) ionisation mode at 70 eV. The quadrupole mass detector, ion source and transfer line temperatures were set, respectively, at 150, 230 and 280°C. Based on the self-established database of Wuhan Meiwei Metabolic Biotechnology Co., LTD., the metabolite content was analyzed by a semi-internal standard quantitative method. Referring to the existing literature reports, the detected substances were classified into carotenoid degradation products, browning reaction products, phenylalanine degradation products and cembranoid diterpenes according to the differences of aroma precursor substances.

### 2.4 Data analysis

The statistical data were analyzed using IBM SPSS Statistics 25 software, and the significance of the mean differences between different treatments was tested by Duncan’s new complex range method (P< 0.05). Pearson’s method was used to calculate the correlation coefficients, and Origin 2024 software was used to generate the correlation heat map. After applying Unit Variance (UV) scaling to the original data, SIMCA-P13.0 econometrics software was used to perform multivariate statistical analysis, including Partial Least Squares Discriminant Analysis (PLS-DA), as well as to generate hierarchical cluster analysis (HCA) graphs and replacement test graphs. The Maiwei cloud platform was used to complete annotation of the KEGG enrichment pathways.

## 3 Results

### 3.1 Selenium content

**Figure 1:**
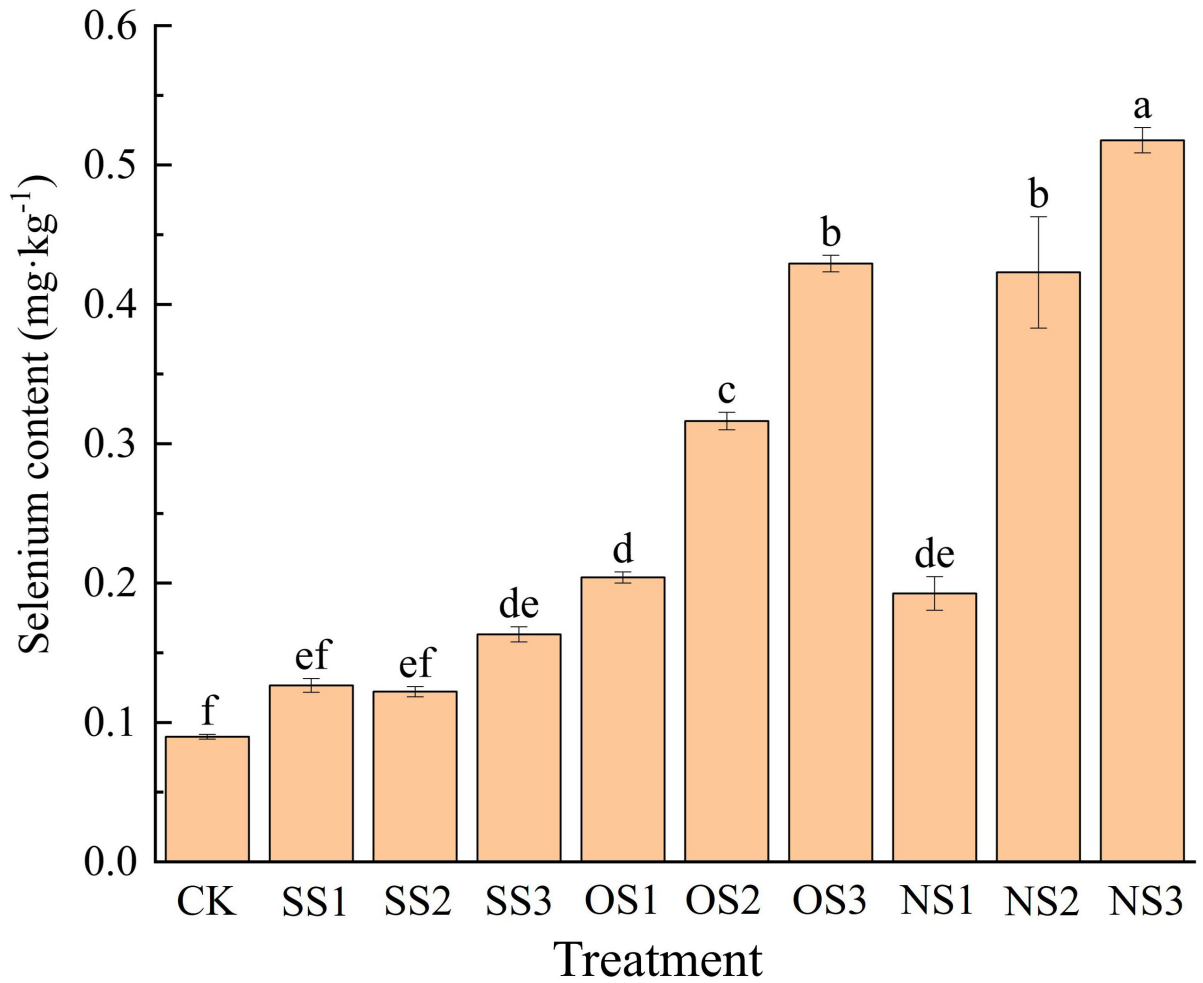
Selenium content under CK, SS1, SS2, SS3, OS1, OS2, OS3, NS1, NS2, NS3. Different letters denote significant differences according to student’s t-test at P< 0.05. CK, no fertilizer; SS1, 5.24 g·hm^-2^ sodium selenite; SS2, 10.48 g·hm^-2^ sodium selenite; SS3, 15.72 g·hm^-2^ sodium selenite; OS1, 5.24 g·hm^-2^ organic selenium fertilizer; OS2, 10.48 g·hm^-2^ organic selenium fertilizer; OS3, 15.72 g·hm^-2^ organic selenium fertilizer; NS1, 5.24 g·hm^-2^ nano-selenium fertilizer; NS2, 10.48 g·hm^-2^ nano-selenium fertilizer; NS3, 15.72 g·hm^-2^ nano-selenium fertilizer.

As shown in **Figure 1**, for the same type of selenium fertilizer, the selenium content increased as the application amount increased, while at the same application rate, selenium content among different types of selenium fertilizers followed the trend: nano-selenium > organic selenium > inorganic selenium.

The selenium content in SS1 and SS2 treatments was not significantly different from CK, whereas the selenium content under the SS3 treatment was 77.78% higher than CK. For the organic selenium and nano-selenium treatments, the difference in selenium content between OS1 and NS1 treatments was not significant, which were 1.22 times and 1.11 times higher than CK, respectively. Compared with CK, the selenium content in the upper leaves under the OS2 and NS2 treatments increased by 2.55 times and 3.67 times, respectively. The OS3 and NS3 treatments resulted in 3.76 times and 4.78 times increases, respectively.

It can be seen that as the selenium application rate increased, the selenium content in the upper leaves under the nano-selenium treatment was significantly higher than that under the organic selenium treatment at the same application rate, with the NS3 treatment resulting in the highest content. Therefore, among the three different types of selenium fertilizer, the absorption efficiency of nano-selenium was the highest, followed by organic selenium and inorganic selenium.

### 3.2 NVOAs

#### 3.2.1 Total amount

**Figure 2:**
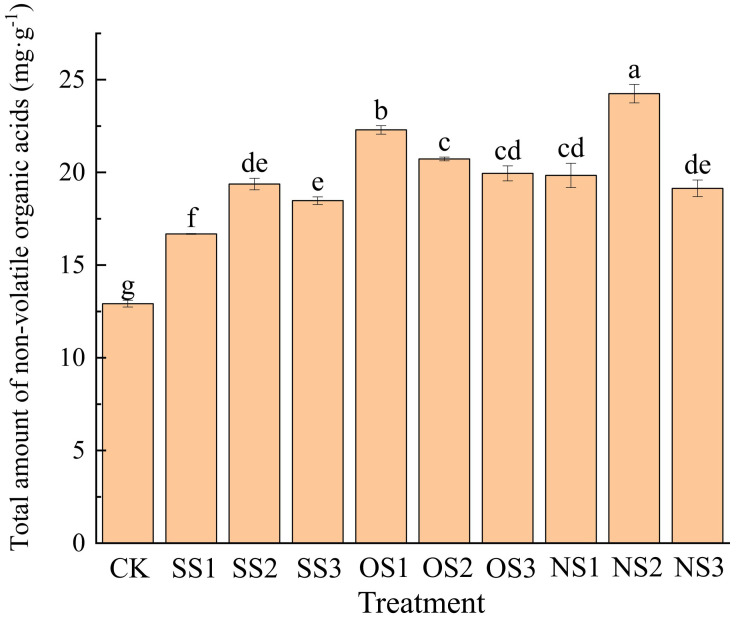
NVOAs content under CK, SS1, SS2, SS3, OS1, OS2, OS3, NS1, NS2, NS3. Different letters denote significant differences according to student’s t-test at P< 0.05.

A total of 44 types of NVOAs were identified and quantified from the upper leaves after flue-curing under different selenium treatments. The total amount of NVOAs for each treatment is shown in **Figure 2**. It can be seen that the application of selenium has significantly increased the NVOAs content, with all treatments showing significantly higher levels than the control (CK).

Among these treatments, the NS2 treatment resulted in the highest, with total NVOAs increasing by 87.69% compared with CK. In the inorganic selenium treatments, the total amount of NVOAs in flue-cured leaves under the SS1 treatment was the lowest, showing an increase of only 29.10% compared with CK. In the organic selenium treatments, the OS1 treatment resulted in the highest amount of NVOAs in flue-cured leaves, with an increase of 72.52% compared with CK. In the nano-selenium treatments, the NS2 treatment resulted in the highest amount.

At the same application rate levels of different selenium fertilizer types, the total NVOAs content in leaves was highest at the second level for inorganic selenium and nano-selenium treatments, whereas it peaked at the first level for the organic selenium treatment. According to the results of selenium content in flue-cured leaves from the different selenium fertilizer treatments, although there was no significant difference in selenium content between the OS3 treatment and NS2 treatments, a significant difference in NVOAs content was observed. This difference may be related to the morphology and conversion efficiency of different types of selenium fertilizers in leaves after absorption.

The component and contents of NVOAs detected are shown in **Table 2**. Among the 44 NVOAs detected, 9 were down-regulated in each treatment: gallic acid, pyruvate, salicylic acid, succinic acid, DL-3-phenyllactic acid, 2-hydroxy-2-methylbutyric acid, kynurenine and methylsuccinic acid. As can be seen from the table, malic acid was the highest, with the highest increase observed under the NS2 treatment, which was 95.46% higher than CK. This substantial increase may be the main reason for the highest total amount of NVOAs in NS2. Except in CK and SS1 significant increment in Cryptochlorogenic acid was in response to all other treatments. In addition, ethylmalonic acid concentration showed a significant increment in response. The contents of the other NVOAs increased to varying degrees after treatment with selenium. Therefore, in order to further identify the regular pattern of NVOAs in which selenium is concentrated, metabolomics analysis were used.

**Table 2:** Quantitative results of non-volatile organic acids in the upper-leaf flue-cured tobacco.

Substance(μg·g^-1^)	CK	SS1	SS2	SS3	OS1	OS2	OS3	NS1	NS2	NS3
Malic acid(mg·g^-1^)	11.68 ± 0.19g	15.38 ± 0.03f	17.95 ± 0.29de	17.04 ± 0.20e	20.90 ± 0.22b	19.30 ± 0.11c	18.57 ± 0.39cd	18.48 ± 0.66cd	22.83 ± 0.49a	17.73 ± 0.42de
Azelaic acid	1.56 ± 0.02g	1.83 ± 0.03f	2.84 ± 0.03c	4.48 ± 0.02a	3.11 ± 0.02b	4.41 ± 0.02a	2.24 ± 0.05e	2.14 ± 0.02e	2.92 ± 0.06c	2.68 ± 0.03d
Cinnamic acid	1.05 ± 0.02g	1.15 ± 0.01e	1.19 ± 0.01d	1.17 ± 0.01de	1.16 ± 0.01e	1.24 ± 0.01c	1.53 ± 0.01b	1.19 ± 0.01d	1.69 ± 0.01a	1.10 ± 0.01f
Cis-aconitic acid	60.63 ± 0.39e	63.41 ± 1.22e	76.26 ± 1.13b	62.86 ± 0.80e	70.36 ± 1.19d	74.81 ± 1.00bc	70.91 ± 1.97d	75.28 ± 0.87bc	72.24 ± 1.38cd	84.10 ± 1.27a
Crtraconic acid	19.00 ± 2.18d	33.27 ± 1.19b	41.53 ± 1.80a	34.37 ± 0.83b	27.84 ± 2.84c	24.41 ± 0.13c	23.04 ± 0.81cd	27.57 ± 0.31c	25.08 ± 1.56c	27.46 ± 1.17c
Cryptochlorogenic acid	658.19 ± 12.97b	662.25 ± 14.75b	750.22 ± 9.38a	784.15 ± 8.40a	750.97 ± 33.18a	745.38 ± 26.46a	738.25 ± 33.00a	722.5 ± 17.27ab	771.99 ± 14.62a	749.23 ± 28.77a
Ferulic acid	3.78 ± 0.02h	4.02 ± 0.01f	4.97 ± 0.01d	5.08 ± 0.01c	4.97 ± 0.01d	4.88 ± 0.01e	5.08 ± 0.01c	5.17 ± 0.01a	5.12 ± 0.01b	3.87 ± 0.01g
Gallic acid	0.11 ± 0.01ab	0.10 ± 0.01b	0.10 ± 0.01b	0.08 ± 0.01c	0.10 ± 0.01b	0.11 ± 0.01ab	0.08 ± 0.02c	0.07 ± 0.01c	0.10 ± 0.01b	0.12 ± 0.05a
Neochlorogenic acid	143.04 ± 1.22d	171.53 ± 6.93bc	167.98 ± 5.04bc	181.78 ± 5.16ab	178.81 ± 3.61ab	194.89 ± 5.61a	167.17 ± 4.72bc	155.59 ± 0.60cd	170.45 ± 0.67bc	189.00 ± 1.70a
Pyruvate	8.00 ± 0.35a	5.34 ± 0.11cd	6.12 ± 0.14bc	4.47 ± 0.05e	5.87 ± 0.07c	5.98 ± 0.09c	5.83 ± 0.15cd	6.59 ± 0.08b	5.62 ± 0.23cd	3.58 ± 0.08f
Shikimic acid	2.54 ± 0.02g	2.62 ± 0.05f	2.62 ± 0.01f	3.56 ± 0.02b	3.24 ± 0.01d	2.72 ± 0.02e	3.17 ± 0.01d	3.81 ± 0.01a	3.43 ± 0.01c	2.70 ± 0.06e
Tartaric acid	69.61 ± 0.72cd	72.8 ± 0.51ab	67.97 ± 0.79d	67.49 ± 1.03d	63.79 ± 0.42e	68.10 ± 1.58d	72.15 ± 1.06abc	74.83 ± 0.78a	70.56 ± 1.02bcd	71.21 ± 1.10bc
Carnosic acid	0.54 ± 0.02e	0.76 ± 0.02de	0.93 ± 0.11cd	1.45 ± 0.12a	0.72 ± 0.02de	1.23 ± 0.09ab	1.06 ± 0.13c	0.66 ± 0.01de	1.42 ± 0.11ab	0.68 ± 0.09de
Caffeic acid	8.77 ± 0.03h	9.44 ± 0.07g	12.74 ± 0.10c	14.31 ± 0.08b	13.88 ± 0.17b	10.91 ± 0.19e	15.08 ± 0.11a	12.14 ± 0.15d	13.92 ± 0.26b	10.09 ± 0.19f
3-Hydroxymethylglutaric acid	14.51 ± 0.52bc	14.02 ± 0.59c	15.84 ± 0.52b	14.35 ± 0.11c	13.39 ± 0.33c	17.39 ± 0.16a	15.8 ± 0.08b	15.82 ± 0.41b	17.57 ± 0.83a	18.04 ± 0.19a
4-Coumaric acid	1.49 ± 0.02f	1.81 ± 0.06d	1.82 ± 0.04d	1.88 ± 0.02cd	2.25 ± 0.03a	2.09 ± 0.03b	1.97 ± 0.02c	1.80 ± 0.02d	1.68 ± 0.04e	1.59 ± 0.04ef
4-Hydroxyhippuric acid	0.97 ± 0.03e	1.59 ± 0.01b	1.27 ± 0.07cd	1.46 ± 0.02bc	1.24 ± 0.04d	1.89 ± 0.02a	1.55 ± 0.06b	1.29 ± 0.06cd	1.66 ± 0.15b	1.58 ± 0.01b
Ethylmalonic acid	3.11 ± 0.24b	3.54 ± 0.16ab	3.34 ± 0.1ab	3.44 ± 0.16ab	3.07 ± 0.10b	3.41 ± 0.14ab	3.44 ± 0.06ab	3.06 ± 0.06b	3.79 ± 0.15a	3.50 ± 0.21ab
Fumaric acid	69.92 ± 0.93b	74.98 ± 1.14a	72.05 ± 0.33ab	65.18 ± 1.61c	58.15 ± 0.99de	61.07 ± 0.47d	57.36 ± 1.69ef	54.14 ± 1.75f	50.68 ± 1.03g	41.00 ± 0.48h
Glutaric acid	2.74 ± 0.13ab	2.47 ± 0.13b	2.89 ± 0.15ab	2.28 ± 0.17b	2.47 ± 0.09b	2.57 ± 0.30b	2.53 ± 0.24b	2.48 ± 0.21b	2.91 ± 0.13ab	3.27 ± 0.29b
Lactic acid	33.72 ± 1.24bc	28.62 ± 0.67d	30.04 ± 2.05cd	34.88 ± 1.27ab	38.06 ± 0.37a	30.57 ± 0.64bcd	29.98 ± 1.35cd	30.68 ± 1.22bcd	31.78 ± 1.70bcd	27.78 ± 2.05d
Pantothenic acid	15.68 ± 0.15f	16.30 ± 0.14f	19.53 ± 0.75cd	18.83 ± 0.70de	17.83 ± 0.32e	22.82 ± 0.49b	20.21 ± 0.09c	17.59 ± 0.16e	24.98 ± 0.48a	21.71 ± 0.19b
Pyroglutamic acid	15.11 ± 0.16e	18.87 ± 0.57cd	19.44 ± 0.48cd	18.57 ± 0.19d	19.22 ± 0.37cd	20.63 ± 0.43bc	22.19 ± 0.20ab	19.92 ± 1.00cd	23.30 ± 0.43a	22.17 ± 0.95ab
Salicylic acid	3.38 ± 0.07a	2.82 ± 0.08b	2.39 ± 0.06c	2.12 ± 0.04d	2.79 ± 0.06b	2.66 ± 0.01b	2.66 ± 0.04b	2.41 ± 0.06c	2.35 ± 0.09c	2.78 ± 0.04b
Succinic acid	61.03 ± 1.31a	59.34 ± 0.28a	56.19 ± 0.13b	51.22 ± 0.14c	59.22 ± 1.46a	51.95 ± 1.45c	47.20 ± 1.69d	54.21 ± 0.48bc	46.88 ± 0.10de	44.16 ± 0.50e
4-Aminobutyric acid	26.17 ± 0.38h	31.05 ± 0.67g	43.75 ± 0.48d	39.48 ± 1.45e	35.87 ± 0.21f	45.12 ± 0.13cd	47.12 ± 1.41c	52.69 ± 1.81b	45.92 ± 0.83cd	59.95 ± 0.66a
2-Hydroxyisovaleric acid	0.14 ± 0.01f	0.18 ± 0.02e	0.19 ± 0.05de	0.23 ± 0.01ab	0.18 ± 0.01e	0.22 ± 0.01bc	0.20 ± 0.01cde	0.21 ± 0.01bcd	0.26 ± 0.01a	0.19 ± 0.01de
Oleanic acid	0.45 ± 0.02c	0.48 ± 0.02c	0.67 ± 0.03b	0.79 ± 0.03a	0.78 ± 0.02a	0.75 ± 0.04a	0.76 ± 0.04a	0.6 ± 0.02b	0.85 ± 0.05a	0.59 ± 0.01b
Aminobenzoic acid	0.12 ± 0.01d	0.14 ± 0.05ab	0.15 ± 0.01a	0.14 ± 0.02ab	0.13 ± 0.02bc	0.14 ± 0.01ab	0.14 ± 0.01ab	0.13 ± 0.01bc	0.13 ± 0.01cd	0.13 ± 0.01bc
2-Hydroxyphenylacetic acid	0.03 ± 0.01b	0.03 ± 0.02b	0.03 ± 0.01b	0.05 ± 0.01a	0.05 ± 0.01a	0.03 ± 0.01b	0.03 ± 0.01b	0.03 ± 0.01b	0.05 ± 0.01a	0.03 ± 0.01b
3-Hydroxyisovaleric acid	0.77 ± 0.01f	0.89 ± 0.03e	1.05 ± 0.02b	0.93 ± 0.05de	0.93 ± 0.01de	0.99 ± 0.02bcd	1.03 ± 0.04bc	1.13 ± 0.01a	1.13 ± 0.01a	0.97 ± 0.01cd
3-Methyladipic acid	0.11 ± 0.01c	0.12 ± 0.01c	0.13 ± 0.01bc	0.16 ± 0.01c	0.14 ± 0.01bc	0.18 ± 0.01a	0.14 ± 0.01b	0.16 ± 0.01b	0.13 ± 0.01bc	0.11 ± 0.01c
3-Phenyllactic acid	2.77 ± 0.04a	2.41 ± 0.06b	2.12 ± 0.01c	1.81 ± 0.05d	1.65 ± 0.04e	1.77 ± 0.04d	1.83 ± 0.02d	1.85 ± 0.01d	1.89 ± 0.05d	1.44 ± 0.02f
Hippuric acid	0.30 ± 0.01f	0.32 ± 0.01ef	0.34 ± 0.01e	0.35 ± 0.01d	0.37 ± 0.01c	0.40 ± 0.01b	0.41 ± 0.02b	0.40 ± 0.02b	0.45 ± 0.01a	0.42 ± 0.01b
Hydroxyphenyllactic acid	1.22 ± 0.05bc	1.30 ± 0.06b	1.43 ± 0.01a	1.07 ± 0.08d	1.27 ± 0.03bc	1.11 ± 0.02d	1.08 ± 0.03d	1.12 ± 0.02cd	1.09 ± 0.01d	1.26 ± 0.01b
Indole-3-acetic acid	0.14 ± 0.01b	0.13 ± 0.02c	0.13 ± 0.01b	0.08 ± 0.01g	0.15 ± 0.02a	0.09 ± 0.01de	0.10 ± 0.05c	0.09 ± 0.01f	0.10 ± 0.02cd	0.14 ± 0.01b
Kynurenic acid	5.19 ± 0.12f	6.02 ± 0.06e	7.15 ± 0.01b	6.35 ± 0.06de	6.77 ± 0.09bc	7.63 ± 0.06a	7.02 ± 0.04b	7.02 ± 0.06b	7.12 ± 0.31b	6.49 ± 0.04cd
Kynurenine	0.30 ± 0.02a	0.23 ± 0.01b	0.22 ± 0.01bc	0.19 ± 0.01cd	0.14 ± 0.01e	0.14 ± 0.01e	0.10 ± 0.01f	0.16 ± 0.01e	0.17 ± 0.01de	0.09 ± 0.01f
Taurine	0.04 ± 0.01g	0.04 ± 0.01ef	0.05 ± 0.00c	0.06 ± 0.01b	0.04 ± 0.01fg	0.05 ± 0.01e	0.06 ± 0.01a	0.05 ± 0.01de	0.05 ± 0.01de	0.05 ± 0.01cd
2-hydroxy-2-methylbutyric acid	1.27 ± 0.02a	1.19 ± 0.02b	1.20 ± 0.01ab	1.13 ± 0.03b	1.03 ± 0.04c	1.03 ± 0.03c	0.98 ± 0.02cd	0.95 ± 0.01d	0.91 ± 0.02d	0.93 ± 0.03d
2-methylsuccinic acid	0.88 ± 0.01a	0.73 ± 0.01b	0.65 ± 0.01c	0.60 ± 0.01d	0.58 ± 0.02de	0.55 ± 0.01e	0.46 ± 0.01f	0.54 ± 0.01e	0.49 ± 0.03f	0.33 ± 0.02g
4-hydroxybenzoic acid	1.63 ± 0.02g	1.74 ± 0.03f	2.04 ± 0.03e	2.11 ± 0.01d	2.24 ± 0.02c	2.33 ± 0.01b	2.14 ± 0.02d	2.29 ± 0.02bc	2.58 ± 0.01a	2.55 ± 0.02a
4-hydroxyphenylacetic acid	0.37 ± 0.01de	0.40 ± 0.01d	0.49 ± 0.02c	0.48 ± 0.01c	0.49 ± 0.02c	0.54 ± 0.01b	0.64 ± 0.02a	0.50 ± 0.02c	0.62 ± 0.01a	0.36 ± 0.01e
5-hydroxymethyl-2-furoic acid	0.74 ± 0.01c	1.03 ± 0.04a	0.81 ± 0.04bc	0.79 ± 0.04bc	0.86 ± 0.03b	0.75 ± 0.03c	0.82 ± 0.01bc	0.60 ± 0.01d	0.78 ± 0.05bc	0.78 ± 0.03bc

Data are presented as mean ± SD (n = 3)). Different letters in the same row indicate significant differences among different treatments at 0.05 level. CK, no fertilizer; SS1, 5.24 g·hm^-2^ sodium selenite; SS2, 10.48 g·hm^-2^ sodium selenite; SS3, 15.72 g·hm^-2^ sodium selenite; OS1, 5.24 g·hm^-2^ organic selenium fertilizer; OS2, 10.48 g·hm^-2^ organic selenium fertilizer; OS3, 15.72 g·hm^-2^ organic selenium fertilizer; NS1, 5.24 g·hm^-2^ nano-selenium fertilizer; NS2, 10.48 g·hm^-2^ nano-selenium fertilizer; NS3, 15.72 g·hm^-2^ nano-selenium fertilizer.

#### 3.2.2 Metabolomics analysis of NVOAs

To further investigate the specific effects of selenium on NVOAs, the results obtained from different selenium application treatments were subjected to UV scaling, followed by partial Least Squares Discrimination Analysis (PLS-DA) and hierarchical cluster analysis (HCA).

**Figure 3:**
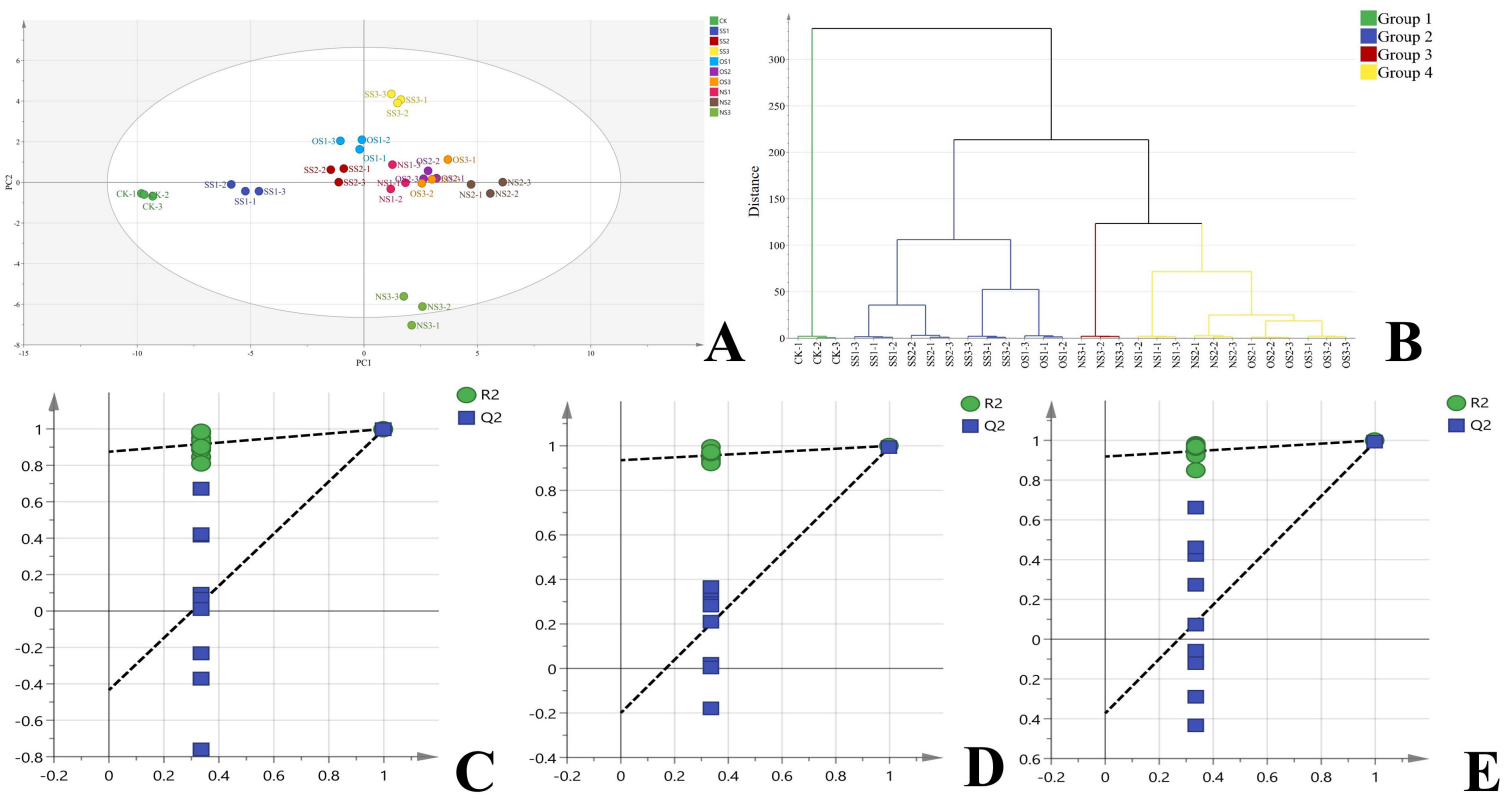
Analysis of NVOAs in the upper-leaf flue-cured tobacco. **(A)** PLS-DA scatter plot based on 44 NVOAs from different treatment; **(B)** Hierarchical clustering analysis based on 44 NVOAs from different treatment; **(C)** OPLS-DA permutation test plot of CK vs SS3; **(D)** OPLS-DA permutation test plot of CK vs OS3 **(E)** OPLS-DA permutation test plot of CK vs NS3.

Based on the abundance of the 44 NVOAs, all samples were divided into four clusters (**Figures 3A, B**). Cluster 1 included CK (selenium content: 0.09 mg·kg^-1^); Cluster 2 included SS1, SS2, SS3, and OS1 treatments (selenium content: 0.12–0.20 mg·kg^-1^); Cluster 3 included NS3 treatments (selenium content: 0.52 mg·kg^-1^); and Cluster 4 included OS2, NS2, OS3, and NS1 treatments (selenium content: 0.32–0.42 mg·kg^-1^). The clustering results showed that the NVOAs profiles of flue-cured leaves varied according to selenium content. Based on the stratification results, we selected CK, SS3, OS3 and NS3, which had significant differences in NVOAs between groups, for subsequent differential marker screening and analysis. The four selected treatments were divided into three comparison groups, namely CKvsSS3 (Group 1), CKvsOS3 (Group 2), and CKvNSS3 (Group 3). The three comparison groups were further subjected to OPLS-DA analysis. The resulting OPLS-DA model had an R^2^X of 0.887, an R^2^Y of 0.991, and a Q^2^ of 0.969. Replacement tests (n=200) were conducted on each group, and the results are shown in **Figure 3C**, **Figure 3D** and **Figure 3E**. The intercept of the Q^2^ and the vertical coordinate in the figure were all less than 0, proving that the model was valid. VIP > 1 and P< 0.05 were used as thresholds to screen for differential markers. There were 33, 34, and 27 differential markers identified in the three comparison groups, respectively.

**Figure 4:**
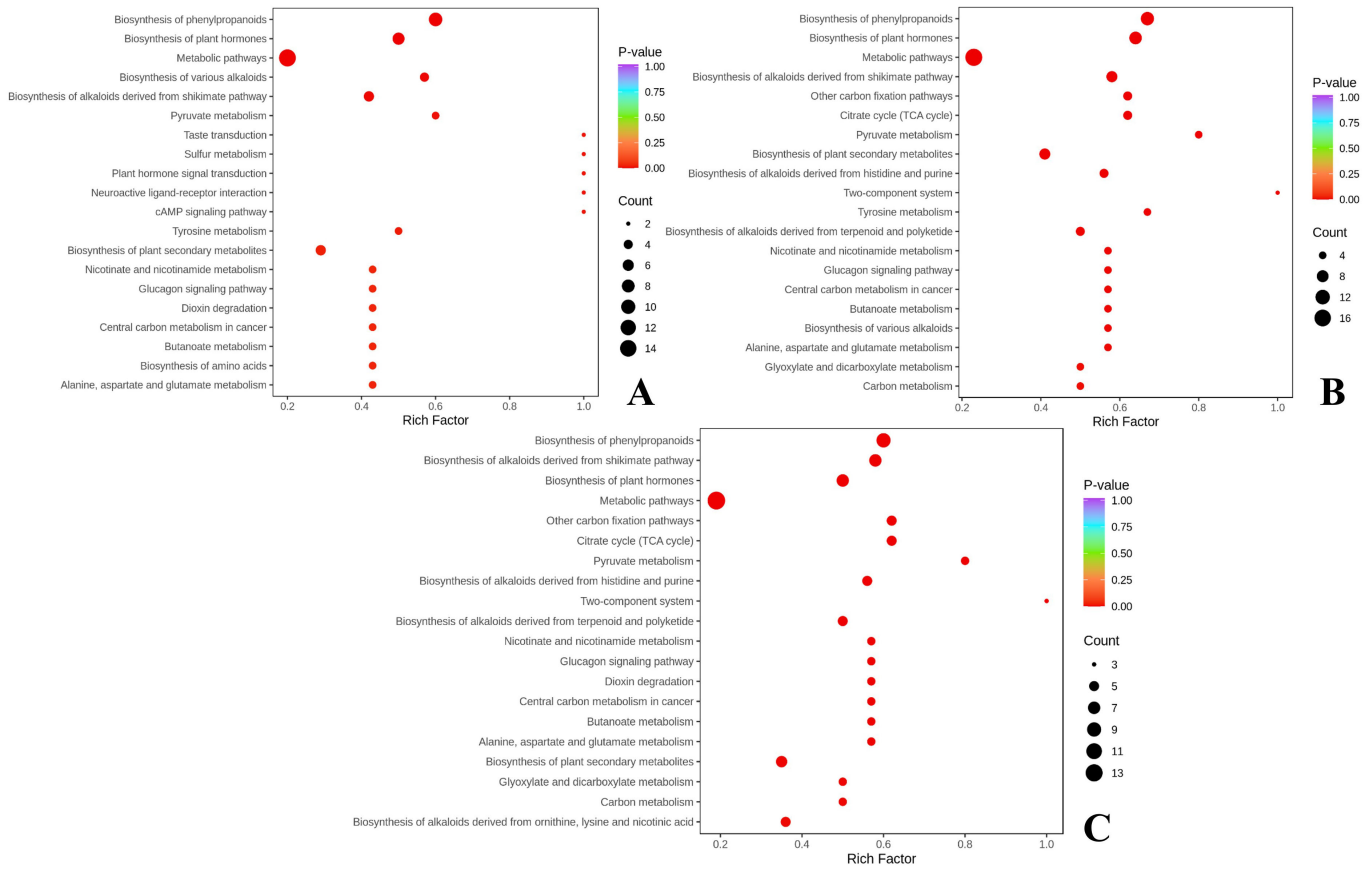
Enrichment analysis of metabolic pathways of differential NVOAs. **(A)** CK vs SS3; **(B)** CK vs OS3; **(C)** CK vs NS3.

The common differential markers screened by the three comparison groups were mapped to the KEGG pathway for annotation and pathway enrichment analysis. The metabolic pathways with significant enrichment were identified using a threshold value of P< 0.05, as shown in **Figure 4**. The biosynthesis of phenylpropanoids was the most significantly enriched. Nine NVOAs were significantly enriched in this pathway across the three comparison groups, including pyruvate, gallic acid, shikimic acid, salicylic acid, cinnamic acid, 4-coumaric acid, ferulic acid, neochlorogenic acid, and cryptochlorogenic acid.

#### 3.2.3 Correlation analysis of DEM of NVOAs and differential metabolic pathways

The pathway with the most numerous of organic acid differential markers and the lowest P-value, namely, the biosynthesis of phenylpropanoids pathway, was selected for further analysis. This pathway begins with pyruvate, which is metabolized by shikimic acid to form phenylalanine. Phenylalanine is subsequently transformed and degraded into cinnamic acid, p-coumaric acid, chlorogenic acid, and various phenols and their precursor substances, which are further converted into benzyl alcohol, phenylethanol, and a series of phenylalanine-derived neutral aroma substances.

**Figure 5:**
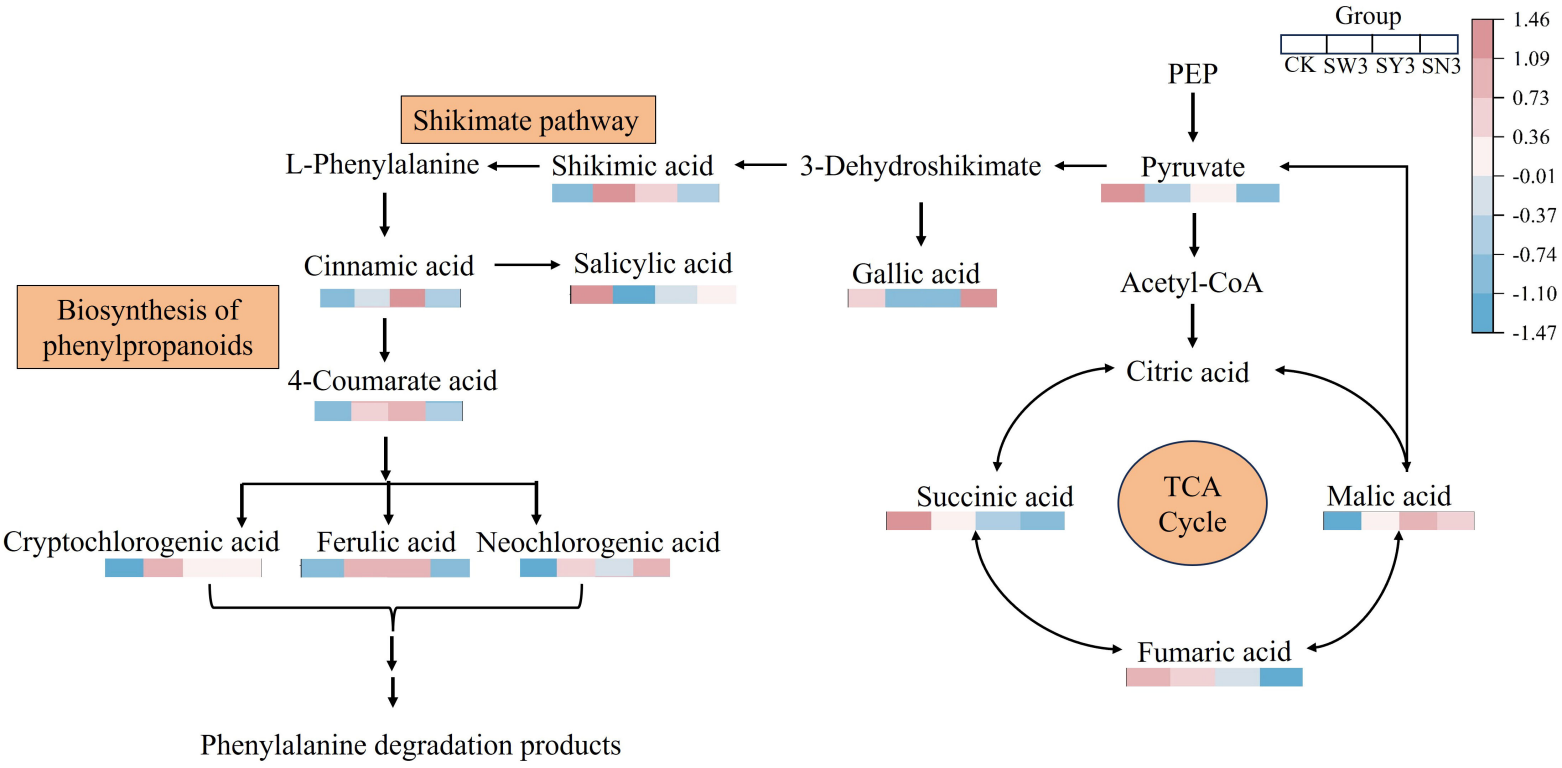
Differential NVOAs in the biosynthesis of phenylpropanoids pathway. The content of the substance in the figure is standardized by Z-score, distinguished by different colors. The color of the square (from blue to red) indicates the significant level of the NVOAs in the data. Each square represents CK, SS3, OS3 and NS3 from left to right in sequence.

The upstream and downstream relationships of the nine NVOAs enriched in the biosynthesis of phenylpropanoids pathway mentioned above are shown in **Figure 5**. It can be seen that the content of pyruvate was significantly down-regulated in each selenium application treatment, while its downstream products, shikimic acid and cinnamic acid, were significantly up-regulated. This indicated that the reduction in pyruvate content was due to its increased conversion into downstream substances rather than a decrease in its synthesis. During the conversion of pyruvate to phenylalanine, branch products such as gallic acid and salicylic acid were significantly down-regulated compared with CK, while the downstream products of phenylalanine were up-regulated compared with CK.

The contents of various phenylalanine-derived neutral aroma substances detected in the current study are shown in **Table 3**. It can be seen that all components, except benzeneacetaldehyde, had significant changes between treatments. Compared with CK, all other substances, except benzoic acid, were significantly increased. Among phenylalanine degradation products, benzyl alcohol accounted for the largest proportion, with the highest content observed under the NS1 treatment, which was 36.03% higher than CK. Acetophenone had the lowest proportion, with the highest content observed under the OS3 treatment, which was 40.00% higher than CK. The highest content of 4-hydroxy-phenyl ethanol was observed under the SS3 treatment, which was 16.67% higher than CK.

**Table 3:** Phenylalanine degradation products in the upper-leaf flue-cured tobacco.

Treatment	Benzoic acid(μg·kg^-1^)	Benzyl alcohol(μg·kg^-1^)	Acetophenone(μg·kg^-1^)	Benzeneacetaldehyde(μg·kg^-1^)	4-Hydroxyphenylethanol(μg·kg^-1^)
CK	0.60 ± 0.04a	4.58 ± 0.54bc	0.10 ± 0.02bc	0.25 ± 0.04ab	0.54 ± 0.04b
SS1	0.54 ± 0.01b	5.25 ± 0.12ab	0.14 ± 0.01ab	0.25 ± 0.07ab	0.60 ± 0.06ab
SS2	0.49 ± 0.01c	5.36 ± 0.36ab	0.13 ± 0.01ab	0.29 ± 0.06ab	0.56 ± 0.09ab
SS3	0.48 ± 0.05c	5.43 ± 0.13ab	0.07 ± 0.01c	0.33 ± 0.01ab	0.65 ± 0.06a
OS1	0.44 ± 0.01cd	4.90 ± 0.17b	0.12 ± 0.01ab	0.23 ± 0.01b	0.54 ± 0.02ab
OS2	0.44 ± 0.01cd	4.89 ± 0.42b	0.12 ± 0.01ab	0.33 ± 0.03ab	0.63 ± 0.06a
OS3	0.40 ± 0.01de	5.28 ± 0.12ab	0.14 ± 0.01a	0.30 ± 0.01ab	0.55 ± 0.03ab
NS1	0.41 ± 0.01de	6.23 ± 0.16a	0.12 ± 0.02ab	0.32 ± 0.01ab	0.53 ± 0.01ab
NS2	0.38 ± 0.01e	5.58 ± 0.36ab	0.09 ± 0.02c	0.34 ± 0.02ab	0.55 ± 0.05ab
NS3	0.39 ± 0.03de	3.89 ± 0.36c	0.05 ± 0.01d	0.36 ± 0.03a	0.44 ± 0.04b

Data are presented as mean ± SD (n = 3)). Different letters in the same column indicate significant differences among different treatments at 0.05 level.

**Figure 6:**
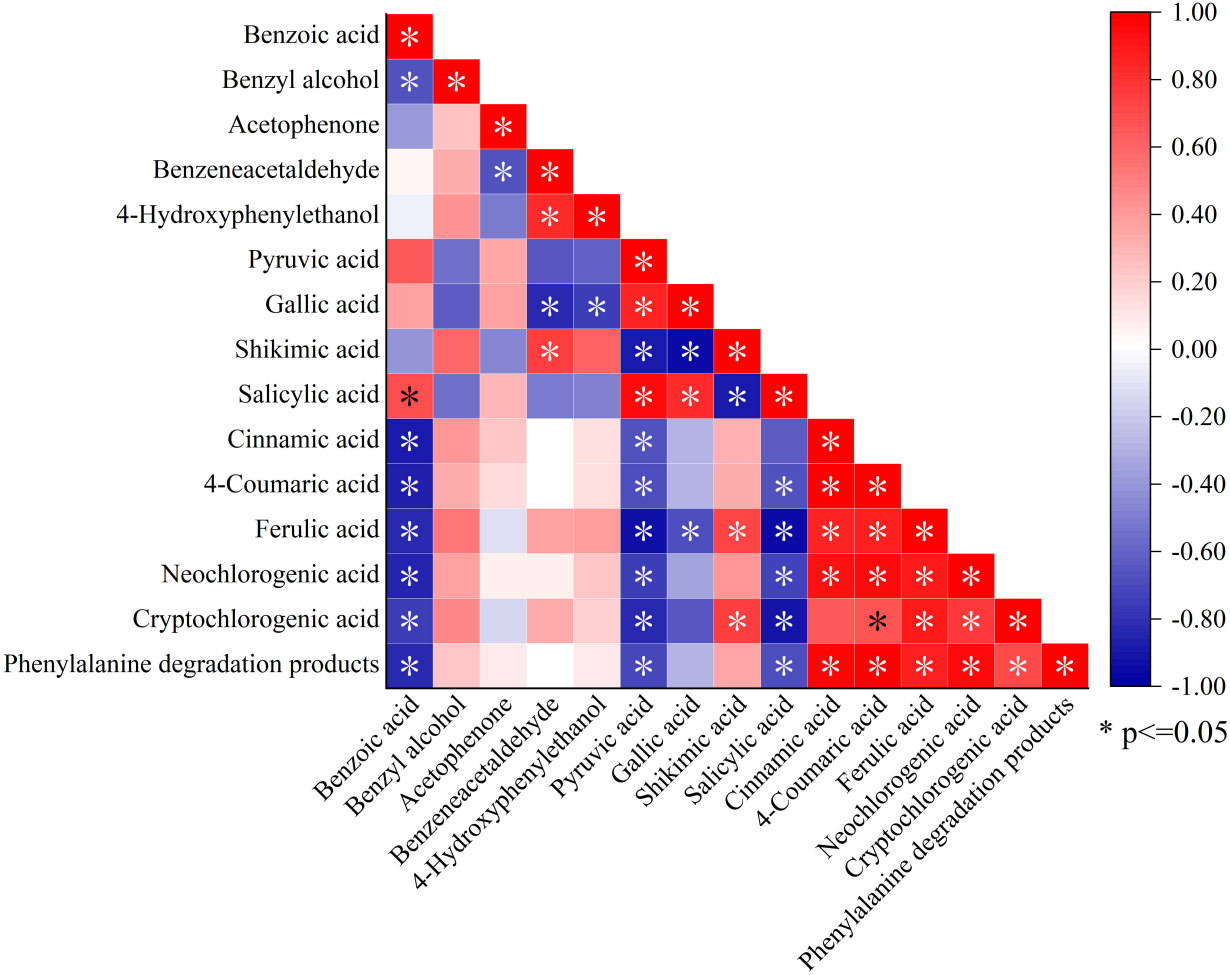
Correlation analysis between phenylalanine degradation products and NVOAs associated with the biosynthesis of phenylpropanoids pathway. *P<0.05.

Correlation analysis between the total amount and components of phenylalanine degradation products and NVOAs enriched by the biosynthesis of phenylpropanoids pathway was shown in **Figure 6**. he content of benzeneacetaldehyde, 4-hydroxy-phenylethanol, and phenylalanine degradation products were significantly negatively correlated with pyruvate. In contrast, the contents of phenylalanine degradation products were significantly positively correlated with cinnamic acid, neochlorogenic acid, and cryptochlorogenic acid. Correlations between other components and other NVOAs were not significant, except for benzoic acid. Therefore, it is speculated that phenolic acids produced by the biosynthesis of phenylpropanoids pathway may be an important factor affecting phenylalanine degradation products in neutral aroma substances.

### 3.3 Aroma precursors

As shown in **Table 4**, the contents of total chlorophyll, petroleum ether extract, and total phenol from each selenium application treatment were significantly higher than those of CK. This promoting effect reached its maximum in the OS2 treatment, which resulted in increases of 2.75 times, 1.48 times, and 43.42%, respectively, compared with CK. However, when the dosage was further increased to the OS3 treatment, the promoting effect began to weaken. The carotenoid content was highest in CK, indicating that a low dose of selenium application may promote the degradation of carotenoids in tobacco leaves after flue-curing. In addition, the carotenoid content in the nano-selenium treatment was significantly higher than that in the inorganic selenium and organic selenium treatments. This may be because nano-selenium is more easily absorbed by leaves, resulting in higher selenium content in tobacco leaves, which affects the degradation of carotenoids.

**Table 4:** Aroma precursors substances in the upper-leaf flue-cured tobacco.

Treatment	Chlorophyll a (mg·kg^-1^)	Chlorophyll b (mg·kg^-1^)	Chlorophyll a + Chlorophyll b (mg·kg^-1^)	Carotenoid (mg·kg^-1^)	Phenolic compounds (mg·kg^-1^)	Petroleum ether extract (%)
CK	0.02 ± 0.01b	0.02 ± 0.00c	0.04 ± 0.00cd	0.11 ± 0.05b	60.89 ± 1.79d	5.32 ± 0.15d
SS1	0.02 ± 0.01b	0.03 ± 0.01c	0.05 ± 0.02cd	0.08 ± 0.02ef	72.31 ± 1.37c	10.90 ± 0.94bc
SS2	0.03 ± 0.02b	0.02 ± 0.01c	0.05 ± 0.01cd	0.10 ± 0.01cd	68.00 ± 0.65c	11.87 ± 0.91abc
SS3	0.02 ± 0.01b	0.03 ± 0.01c	0.05 ± 0.01cd	0.08 ± 0.03ef	80.07 ± 2.69b	10.70 ± 0.59c
OS1	0.04 ± 0.01a	0.06 ± 0.02b	0.10 ± 0.02b	0.10 ± 0.01cd	68.09 ± 0.79c	10.43 ± 0.10c
OS2	0.05 ± 0.01a	0.10 ± 0.02a	0.15 ± 0.03a	0.08 ± 0.01f	87.33 ± 0.81a	13.22 ± 0.08a
OS3	0.04 ± 0.01a	0.07 ± 0.01ab	0.11 ± 0.01ab	0.10 ± 0.05c	71.81 ± 1.85c	12.23 ± 0.69abc
NS1	0.02 ± 0.01b	0.03 ± 0.01c	0.05 ± 0.01cd	0.09 ± 0.05ef	69.85 ± 1.54c	11.82 ± 0.51abc
NS2	0.02 ± 0.00b	0.01 ± 0.00c	0.03 ± 0.01d	0.09 ± 0.01de	86.10 ± 2.53a	11.56 ± 0.08abc
NS3	0.04 ± 0.01a	0.04 ± 0.01bc	0.08 ± 0.01bc	0.13 ± 0.05a	78.98 ± 0.91b	12.80 ± 0.46ab

Data are presented as mean ± SD (n = 3). Different letters in the same column indicate significant differences among different treatments at 0.05 level.

### 3.4 Neutral aroma substances

The neutral aroma substances detected in each treatment were analyzed by variance, as shown in **Table 5**. It can be seen that, except for the NS3 treatment, all other treatments showed varying degrees of improvement compared with CK.

**Table 5:** Neutral aroma substances in the upper-leaf flue-cured tobacco.

Treatments	Carotenoid degradation products (μg·kg^-1^)	Brown chemical reaction products (μg·kg^-1^)	Phenylalanine degradation products (μg·kg^-1^)	Cembranoid diterpenes (μg·kg^-1^)	Total Neutral aroma components (μg·kg^-1^)
CK	5.87 ± 0.33cd	2.24 ± 0.20b	5.94 ± 0.60c	1.61 ± 0.22b	15.67 ± 1.22bc
SS1	6.98 ± 0.03bc	2.50 ± 0.07ab	6.69 ± 0.09ab	1.57 ± 0.17b	17.74 ± 0.32ab
SS2	7.37 ± 0.26ab	2.49 ± 0.23ab	6.83 ± 0.51ab	1.68 ± 0.19ab	18.37 ± 0.85ab
SS3	7.97 ± 0.80ab	2.40 ± 0.08ab	6.90 ± 0.28ab	2.11 ± 0.07a	19.37 ± 1.15a
OS1	7.16 ± 0.19ab	2.40 ± 0.08ab	6.15 ± 0.27bc	2.08 ± 0.04a	17.79 ± 0.56ab
OS2	8.49 ± 0.05a	2.62 ± 0.21ab	7.60 ± 0.21a	1.75 ± 0.18ab	20.45 ± 0.85a
OS3	8.22 ± 0.22ab	2.72 ± 0.01a	6.69 ± 0.15ab	1.82 ± 0.01ab	19.46 ± 0.25a
NS1	8.16 ± 0.69ab	2.56 ± 0.02ab	6.38 ± 0.43abc	1.38 ± 0.02b	19.69 ± 0.85a
NS2	7.26 ± 0.20ab	2.40 ± 0.12ab	6.89 ± 0.42ab	1.69 ± 0.08ab	18.25 ± 0.74ab
NS3	5.57 ± 0.52d	2.16 ± 0.15b	5.31 ± 0.48c	0.81 ± 0.16c	13.85 ± 1.28c

Data are presented as mean ± SD (n = 3). Different letters in the same column indicate significant differences among different treatments at 0.05 level.

The carotenoid degradation products in the OS2 treatment were the highest, showing an increase of 443.63% compared with CK, while all other treatments, except for SS1 and NS3, were also higher than CK, significantly. The amount of browning reaction products in the OS3 treatment was the highest, being 21.42% higher than that of CK; however, there were no significant differences between the other treatments and CK. The highest amount of phenylalanine degradation products was observed in the OS2 treatment, showing an increase of 27.94% compared with CK, while all other treatments, except for NS3, were also significantly higher than CK. The SS3 and OS1 treatments showed the highest performance in improving cembranoid diterpenes, with increases of 31.06% and 29.19% compared with CK, respectively. No significant differences were observed among the other treatments, except for NS3. The total amount of neutral aroma substances was the highest in the OS3 treatment, which was 24.19% higher than that in CK.

### 3.5 Correlation analysis of selenium content with NVOAs, aroma precursors and neutral aroma substances

**Figure 7:**
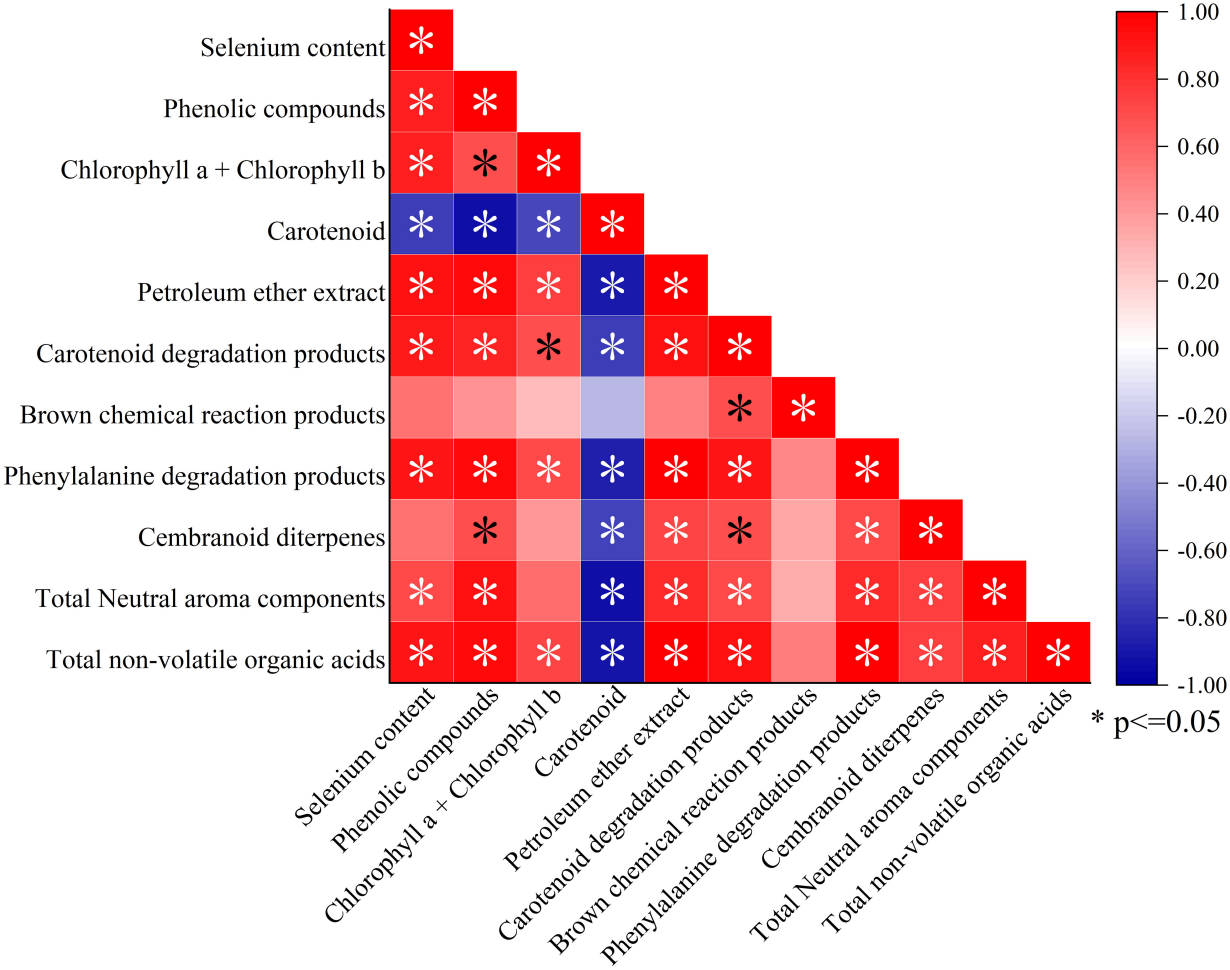
Correlation analysis of selenium content recorded in the leaves with aroma precursors and neutral aroma substances. *P<0.05.

The Pearson correlation analysis of selenium content with NVOAs, aroma precursors, and neutral aroma substances is shown in **Figure 7**. The results show that selenium content was significantly positively correlated with total organic acid content, total phenol content, chlorophyll content, and petroleum ether extract content, while it was significantly negatively correlated with carotenoid content.

In terms of neutral aroma substances, selenium content was significantly positively correlated with carotenoid degradation products, suggesting that selenium application may promote the degradation of carotenoids during the late growth or curing stages of flue-cured tobacco. The total NVOAs were significantly positively correlated with phenolic compounds, chlorophyll, petroleum ether extracts, carotenoid degradation products, phenylalanine degradation products, siparane degradation products, and neutral aroma substances. The correlation analysis between NVOAs and phenylalanine degradation products indicated that phenolic acids in NVOAs, such as cinnamic acid, neochlorogenic acid, and cryptochlorogenic acid, were significantly positively correlated with phenylalanine degradation products.

Therefore, selenium application is likely to promote the entire pathway from pyruvate → dehydroshikimic acid → shikimic acid → phenylalanine → phenylalanine degradation substances by enhancing the conversion of pyruvate to cinnamic acid. This process ultimately promotes the accumulation of phenylalanine degradation products and phenolic substances, which are intermediate products of biosynthesis of phenylpropanoids pathway. The results showed that the content of NVOAs was positively correlated with the content of phenolic substances. Selenium content was significantly positively correlated with the total amount of neutral aroma substances, further confirming that selenium application has an effect on the improvement of neutral aroma substances in tobacco leaves. This effect might be achieved by increasing carotenoid degradation products and phenylalanine degradation products.

## 4 Discussion

Selenium, which is a beneficial trace elements for plant growth, helps stimulate plant development, boost crop yield and quality, improve plant resistance to stress, and promote plant disease and pest resistance (Winkel et al., 2012; Zhou et al., 2025). Based on different forms, selenium fertilizer can be divided into inorganic selenium fertilizer, organic selenium-rich fertilizer, biological selenium-rich fertilizer, nano-selenium-rich fertilizer, slow-release selenium-rich fertilizer, etc. Currently, most studies on selenium application are based on the use of sodium selenite, which is an inorganic selenium fertilizer (Li et al., 2020). Previous studies have demonstrated that, compared with traditional selenium preparations, nano-selenium fertilizer is characterized by its small particle size and large surface area, resulting in stronger biological activity and bioavailability (Lin et al., 2023; Zhao et al., 2018). In the current study, the effects of three selenium fertilizers, sodium selenite, organic selenium-rich fertilizer, and biological nano-selenium, were compared. In each treatment, the selenium content recorded in the leaves was within the range of 0.09–0.52 mg·kg^-1^ depending on the application amount, which was within the appropriate range recognized by the researchers (Cao et al., 2017). From the experimental results (**Table 1**), it can be seen that with the increase of selenium application rate, the selenium content in the tobacco leaves after roasting increases accordingly. However, when comparing at the same dose, the effectiveness of improving selenium content in tobacco leaves followed the order: nano-selenium, organic selenium, and inorganic selenium. This indicates that the utilization rate of selenium fertilizer is the highest for nano-selenium, followed by organic selenium, and the lowest for inorganic selenium. In this experiment, we focused on comparing the effects of different selenium fertilizers on NVOAs in flue-cured tobacco (**Figure 2**). However, by linking the selenium content in tobacco leaves with the total amount of NVOAs, it can be found that there is not a simple linear positive correlation between NVOAs and selenium content. The selenium content reached the highest in NS3, but the total amount of NVOAs was significantly lower than that in NS2 treatment. This indicates that the enhancement effect of selenium on organic acids began to weaken when the selenium content in the leaves was 0.42 mg·kg^-1^. Notably, although the selenium content in OS3 was significantly higher than that in NS1, the same trend was not observed in NVOAs content. This might be because different forms of selenium fertilizer can affect the existence form of selenium in the leaves of flue-cured tobacco (Pei et al., 2025). Meanwhile, we can also observe from the results of the HCA diagram (**Figure 3B**) that although the NVOAs content of NS2 is the highest among all treatments, its NVOAs profile is still very similar to that of OS2, OS3 and NS1, leading to these treatments being clustered into the same group. Previous studies have proved that selenium can be converted into a variety of selenides after being absorbed, transformation, and metabolism by plants (Chao et al., 2022; Gupta and Gupta, 2017). Therefore, whether it is different forms of foliar selenium fertilizers that affect the existence form of selenium in plants after being absorbed, influence the metabolism of NVOAs, and showing different effects in NVOAs profile, still requires further exploration of different selenium components in tobacco.

Phenolic compounds originated from the shikimic acid pathway are an important secondary metabolite, besides responding to the stress resistance function of crops under stress, they are also serve as important aroma precursors (Guedes et al., 2023; Fan et al., 2022). The content of phenolic substances and their degradation products is closely related to the taste, fragrance type, and aroma intensity of flue-cured tobacco. These compounds are the main substances affecting the aroma of tobacco leaves (Fan et al., 2022). The proportion of carotenoid degradation products in the neutral aroma substances is about 8-12%. These compounds belong to the characteristic flavor components of tobacco with a low threshold, good aroma and mild irritation (Zhu et al., 2016; Song et al., 2010). Petroleum ether extract of flue-cured tobacco mainly refers to aroma precursors such as aromatic oil, resinous aldehydes, phospholipids, and paraffin secreted from the glandular hairs of tobacco leaves. It is an important index for evaluating the aroma and taste of flue-cured tobacco (Chen et al., 2019; Sun et al., 2023). This study showed that the selenium fertilizer could significantly increase the content of petroleum ether extract and phenolic substances, as well as the content of chlorophyll in the leaves after flue-curing. The correlation analysis indicated that the carotenoid content in the tobacco leaves after roasting was significantly negatively correlated with the selenium content, and at the same time, in the neutral aroma substance results, the carotenoid degradation products showed a significant increase in the selenium treatment. However, some studies have pointed out that selenium application can increase the carotenoid content during the growth period of flue-cured tobacco (Han et al., 2013). Therefore, it cannot be simply assumed that selenium application can enhance the degradation of carotenoids. Instead, a comprehensive judgment should be made in combination with the activity of enzymes related to the degradation of carotenoids. This indicates that the effects of selenium on plastid pigment components are different, and the specific mechanism needs to be further analyzed in subsequent studies. At the same time, selenium application can effectively increase the total amount of NVOAs in the upper leaves of flue-cured tobacco.

The aroma substances of tobacco leaves are classified as secondary metabolites. The main substances affecting the aroma of tobacco leaves, such as phenols, terpenoids, and alkaloids, are synthesized through three secondary metabolic pathways in plants, namely, the biosynthesis of phenylpropanoids pathway, the isoprene metabolic pathway, and the alkaloid metabolic pathway (Jing et al., 2005). The biosynthesis of phenylpropanoids pathway originates from the phenylalanine, it could be catalyzed by phenylalanine ammonia-lyase (PAL) to form an important intermediate substance, cinnamic acid. With the participation of hydroxylase, acyltransferase, methyltransferase, and other enzymes, a series of reactions, such as hydroxylation, acylation, and methylation, can form caffeic acid, ferulic acid, erucic acid and other phenolic intermediates (Schieber and Wüst, 2020; Skaliter et al., 2022). This process is the main pathway for the synthesis of phenolic substances (McCALLA and NEISH, 1959). By further enrichment annotation of the detected NVOAs through the KEGG pathway, it was found that differential NVOAs were significantly enriched in the biosynthesis of phenylpropanoids pathway. Phenolic acids, including caffeic acid, 4-coumaric acid, ferulic acid, etc., were significantly up-regulated, which was consistent with the results of studies on alfalfa and dragon fruit (Wang et al., 2023; Yu et al., 2023). These substances are further degraded to produce small molecule aroma substances, such as benzoic acid, phenylacetic acid, benzyl alcohol, and phenyl ethanol (Schieber and Wüst, 2020; Xu et al., 2024), which are important sources of neutral aroma substances in tobacco, and contribute significantly to the fruity and fragrant taste of flue-cured tobacco (Niu et al., 2020). The application of selenium fertilizer has significantly increased the content of phenylalanine degradation products (**Table 5**), however, if the influence of selenium application on the flavor and aroma style of flue-cured tobacco is to be further evaluated, it still needs to be verified in subsequent experiments on this basis. We also observed that pyruvate, the precursor substance of the biosynthesis of phenylpropanoids pathway, showed a stronger downward trend with the enhancement of selenium treatment, which is inconsistent with what was observed on broccoli (**Table 2**; **Figure 5**; Liu et al., 2023). We consider that this might be caused by the different doses of selenium application and the inconsistent tolerance of crops to selenium.

In the current study, the total amount of benzeneacetaldehyde, 4-hydroxy-phenylethanol, and phenylalanine degradation products was significantly negatively correlated with pyruvate. Additionally, the contents of phenylalanine degradation products were significantly positively correlated with cinnamic acid, neochlorogenic acid, and cryptochlorogenic acid(**Figure 5**). Therefore, it was considered that phenylalanine degradation products were related to the conversion of these three NVOAs. Through the correlation analysis of aroma precursor substances such as phenolic compounds, plastid pigments, and neutral aroma substances with selenium content, it was found that selenium content was positively correlated with phenolic compounds, petroleum ether extract, phenylalanine degradation products, and carotenoid degradation products, while being significantly negatively correlated with carotenoid content, which is consistent with the pattern observed on pitayas (Wang et al., 2023). Phenolic substances are secondary metabolites produced during the biosynthesis of phenylpropanoids pathway, which is consistent with the experimental results showing that phenolic compounds are positively correlated with the degradation products of phenylalanine.

Therefore, this experiment suggested that the application of selenium can promote the biosynthesis of phenylpropanoids pathway in tobacco, ultimately increasing the content of phenolic acids downstream and leading to the overall up-regulation of phenylalanine degradation products. This may be an important reason for the increase of aroma substances in tobacco leaves after flue-curing. Overall, we believe that selenium fortification of tobacco helps improve its aroma quality.

## 5 Conclusion

In the current study, the effects of selenium application on the content of NVOAs and neutral aroma substances in flue-cured tobacco were investigated at the metabonomics level for the first time. It was found that selenium fertilizers spraying significantly increased the content of neutral aroma substances and NVOAs in the upper leaves after flue-curing. Metabolomics analysis revealed that the regulation of NVOAs in the biosynthesis of phenylpropanoids pathway may be conducive to the accumulation of phenylalanine degradation products. Therefore, we speculated that selenium application could regulate the metabolic pathways of certain NVOAs and enhance the conversion of pyruvate into the phenylpropanoid metabolism pathway thereby promote the accumulation of aroma precursors and neutral aroma substances. Considering the content of NVOAs and the neutral aroma substances, the selenium content range of the treatment with the best performance in this experiment was 0.20–0.42 mg·kg^-1^, which could be used as the recommended selenium content range for selenium strengthening of flue-cured tobacco leaves. In this experiment, the corresponding treatment was organic selenium 5.24–15.72 g·hm^-2^ or nano-selenium 5.24–10.48 g·hm^-2^.

## Data Availability

The original contributions presented in the study are included in the article/**Supplementary Material**. Further inquiries can be directed to the corresponding authors.
