# Supplementary material for: Effects of different selenium fertilizer types and dosages on non-volatile organic acids and aroma substances of flue-cured tobacco
Source: Front Plant Sci. 2025 Aug 22;16:1659004. doi: 10.3389/fpls.2025.1659004 (PMC12412258; doi:10.3389/fpls.2025.1659004)
Supplement: Supplementary file 2 [file Table1.docx]

**Table S1**

**Experimental treatment in 2023**

| Treatment | Selenium fertilizer type | Selenium application |
| --- | --- | --- |
| CK | Water only | none |
| S0 | organic selenium fertilizer | 10.50 g·hm^-2^ |
| S1 | organic selenium fertilizer | 21.00 g·hm^-2^ |
| S2 | organic selenium fertilizer | 31.50 g·hm^-2^ |
| S3 | organic selenium fertilizer | 42.00 g·hm^-2^ |
| S4 | organic selenium fertilizer | 52.50 g·hm^-2^ |

**Table S2**

**Selenium content in tobacco leaves in 2023, different letters denote significant differences according to student’s t-test at P < 0.05**

| Treatment | Upper leaves（mg·kg^-1^） | 中部叶/（mg·kg^-1^） |
| --- | --- | --- |
| CK | 0.15±0.04e | 0.17±0.00e |
| S0 | 0.58±0.01d | 0.53±0.00d |
| S1 | 1.25±0.01c | 1.76±0.05c |
| S2 | 1.45±0.02b | 2.05±0.03b |
| S3 | 2.34±0.05a | 2.49±0.05a |
| S4 | 1.46±0.03b | 1.81±0.02c |
